# Enhanced Production of Gypenoside LXXV Using a Novel Ginsenoside-Transforming β-Glucosidase from Ginseng-Cultivating Soil Bacteria and Its Anti-Cancer Property

**DOI:** 10.3390/molecules22050844

**Published:** 2017-05-19

**Authors:** Chang-Hao Cui, Da Jung Kim, Suk-Chae Jung, Sun-Chang Kim, Wan-Taek Im

**Affiliations:** 1 Intelligent Synthetic Biology Center, 291 Daehak-ro, Yuseong-gu, Daejeon 305-701, Korea; seldoms@163.com (C.-H.C.); scjung@kaist.ac.kr (S.-C.J.); 2 Department of Biological Sciences, Korea Advanced Institute of Science and Technology, 291 Daehak-ro, Yuseong-gu, Daejeon 305-701, Korea; kimdj1404@kaist.ac.kr; 3 KAIST Institute for Biocentury, Korea Advanced Institute of Science and Technology, 291 Daehak-ro, Yuseong-gu, Daejeon 305-701, Korea; 4 Department of Biological Sciences, Hankyong National University, 327 Chungang-no Anseong-si, Kyonggi-do 456-749, Korea

**Keywords:** gypenoside LXXV, gypenoside XVII, ginsenoside, deglycosylation, biotransformation, *Microbacterium* sp. Gsoil 167

## Abstract

Minor ginsenosides, such as compound K, Rg_3_(*S*), which can be produced by deglycosylation of ginsenosides Rb_1_, showed strong anti-cancer effects. However, the anticancer effects of gypenoside LXXV, which is one of the deglycosylated shapes of ginsenoside Rb_1_, is still unknown due to the rarity of its content in plants. Here, we cloned and characterized a novel ginsenoside-transforming β-glucosidase (BglG167b) derived from *Microbacterium* sp. Gsoil 167 which can efficiently hydrolyze gypenoside XVII into gypenoside LXXV, and applied it to the production of gypenoside LXXV at the gram-scale with high specificity. In addition, the anti-cancer activity of gypenoside LXXV was investigated against three cancer cell lines (HeLa, B16, and MDA-MB231) in vitro. Gypenoside LXXV significantly reduced cell viability, displaying an enhanced anti-cancer effect compared to gypenoside XVII and Rb_1_. Taken together, this enzymatic method would be useful in the preparation of gypenoside LXXV for the functional food and pharmaceutical industries.

## 1. Introduction

Ginseng (*Panax ginseng* C.A. Meyer) has been widely used to treat various diseases for thousands of years in Asian countries and, nowadays, is used as a medicinal herb or functional food all over the world [1,2]. Ginsenosides are considered as the main active ingredients responsible for pharmacological activities of the ginseng root, such as anti-cancer, anti-obesity, anti-inflammatory, antioxidant, and hepatoprotective activities [3,4,5,6,7]. The six major ginsenosides (Rb_1_, Rb_2_, Rc, Rd, Re, and Rg_1_) constitute more than 80% of the total ginsenosides in ginseng root, and various minor ginsenosides (Rg_3_(*S*), Rh_2_(*S*), F_2_, compound K (C-K), Rg_2_(*S*), Rh_1_(*S*), F_1_, protopanaxadiol (PPD) and protopanaxatriol (PPT), and gypenoside XVII (GypXVII)) that are deglycosylated from the major ginsenosides exist in smaller amounts in ginseng [8,9,10,11]. Major ginsenoside Rb_1_ is relatively abundant in ginseng, and it can be deglycosylated into rare minor ginsenosides (F_2_, Rg_3_(*S*), Rh_2_(*S*), CK, PPD) which have enhanced anti-cancer activities [12,13].

GypXVII and gypenoside LXXV (GypLXXV), which have primarily been found in *Gynostemma pentaphyllum* are also the deglycosylated products of major ginsenoside Rb_1_ [14]. However, the pharmacological effects of GypXVII and GypLXXV is still unknown, because of their rare contents in *G. pentaphyllum* or *P. ginseng* C.A. Meyer. Although various purification methods were developed to improve the yield of saponins from the raw plants, neither GypXVII nor GypLXXV have been purified enough from raw plants for pharmacological study [15].

In this study, we isolated a novel ginsenoside-transforming β-glucosidase (BglG167b) from a ginseng-cultivating soil bacteria. BglG167b was cloned and overexpressed in *Escherichia coli* and the enzymatic properties and substrate specificities were investigated. This enzyme could hydrolyze various ginsenosides (Rb_1_, Rb_2_, Rc, Rd, Rg_3_(*S*), F_2_, Re, Rg_1_, GypXVII) through various cleavage pathways, and has been applied for the production of GypLXXV from GypXVII at the gram-scale. Eventually, the pure GypLXXV was used for the study of anti-cancer effects against three kinds of cancer cells in vitro.

## 2. Results and Discussion

### 2.1. Isolation and Characterization of Strain Microbacterium *sp.* Gsoil 167

Strain Gsoil 167 which has been isolated from ginseng cultivating soil was confirmed to hydrolyze Rg_1_, Re and Rb_1_ into PPT and PPD, respectively (Data not shown), and was selected for the screening of ginsenoside-transforming enzymes. Phylogenetic analysis based on 16S rRNA gene sequences indicated that the isolate belongs to the genus *Microbacterium* in the phylum *Actinobacteria*, and is most closely related to *Microbacterium luteolum* IFO 15074^T^ (99.9% similarity).

### 2.2. Fosmid Library Construction and Cloning of BglG167b

Genomic DNA from *Microbacterium* sp. Gsoil 167 was isolated by phenol-chloroform extraction and used to generate a fosmid library. β-d-glucosidase activity was detected from a fosmid vector containing an inserted contig (9.4 kb). Twenty-three (23) putative ORFs of longer than 300 codons have been found using an ORF FINDER [16] analysis. Among them, one was homologous to glycoside hydrolase genes in glycoside hydrolase family 3 (GH3). This ORF, termed BglG167b, consisting of 2412 bp encoding 803 amino acids, was amplified via polymerase chain reaction (PCR) and then inserted into the pGEX 4T-1 vector.

### 2.3. Phylogenetic Analysis of BglG167b Sequences

The β-glucosidase (BglG167b) has a predicted molecular mass of 90.3 kDa and a theoretical *pI* value of 4.56 [17]. Analysis of the amino acid sequences of BglG167b indicated that it was 79% identical to the glycoside hydrolase of *Microbacterium paraoxydans* (GenBank number WP_018187396), which belongs to GH3. The enzyme from *M. paraoxydans* has not yet been characterized. The nearest characterized glycoside hydrolase in GH3 in the CAZy database was β-d-glucosidase from *Agrobacterium tumefaciens*, which was 40% identical to BglG167b based on amino acid sequences [18]. The phylogenetic analysis resulting consensus tree is presented in Figure S1. BglG167b clustered within subfamily 5 and formed a separate, well-supported clade (bootstrap of 100) with cellobiase of *Cellulomonas biazotea*, uncultured bacterium β-glucosidase and uncultured rumen bacterium β-glucosidase [19,20,21].

Various ginsenoside-hydrolyzing β-glucosidases in GH3 have been previously characterized, including two β-glucosidases (Bgp1 and Bgp3) from *Microbacterium esteraromaticum*, a β-glucosidase (rApy-H11) from *Bifidobacterium longum* H-1, a β-glucosidase (BGL1) from *Aspergillus niger*, a β-glucosidase (BglAm) from *Actinosynnema mirum*, a β-glucosidase (BgpA) from *Terrabacter ginsenosidimutans*, and a β-glucosidase (BglSk) from *Sanguibacter keddieii* [22,23,24,25,26,27,28,29]. The relationship between BglG167b and these ginsenoside-hydrolyzing β-glucosidases is presented in Figure S1.

### 2.4. Purification of Recombinant BglG167b

**Figure 1:**
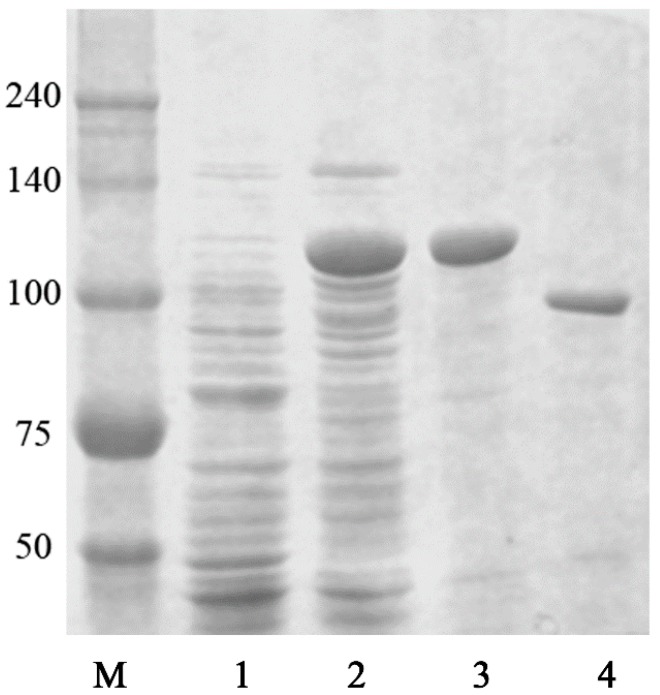
SDS-PAGE analysis of recombinant BglG167b. Lane M, molecular weight standard; lane 1, crude extract of BL21 (DE3) carrying pGEX-BglG167b without induction; lane 2, soluble fraction in crude extract of BL21 (DE3) carrying pGEX-BglG167b after induction; lane 3, GST-BglG167b enzyme fraction after purification with the GST-bind agarose resin; lane 4, purified recombinant BglG167b after cleavage by thrombin2.5. Characterization of recombinant BglG167b.

The GST-fused BglG167b protein was purified using GST-Bind agarose resin. The recombinant enzyme was purified by GST-Bind agarose resin, and then the purified protein was determined using SDS-PAGE analysis (Figure 1). The molecular mass of the GST-BglG167b calculated via an amino acid sequence was 116 kDa, which is similar to the mass detected by SDS-PAGE. In addition, the recombinant GST-BglG167b contains 36.5 ± 1.4% of total soluble proteins in the cell lysate.

**Figure 2:**
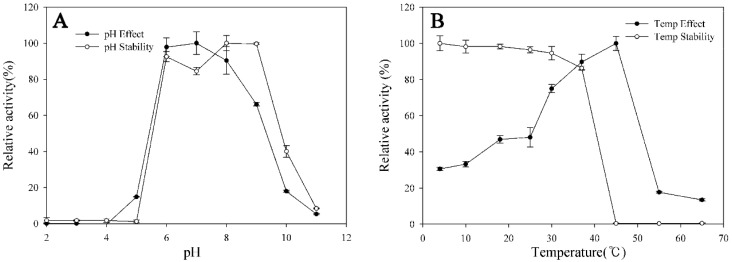
Effects of pH (**A**) and temperature (**B**) on the stability and activity of recombinant BglG167b.

The optimum temperature and pH were characterized using purified GST-BglG167b. The optimal temperature activity was 45 °C; at 30 °C and 37 °C, the enzyme has 75.0% and 89.8% of relative activity, while thermo-stability was decreased significantly from 45 °C, and not detected after incubation at 45 °C for 2 h (Figure 2A). The enzyme was stable at temperatures lower than 30 °C, and about 86.4% of the activity was lost after incubation at 37 °C for 2 h (Figure 2A). The optimal temperature for the BglG167b activity was similar to the β-glucosidase isolated from *T. ginsenosidimutans* (45 °C) [22]. BglG167b had optimal pH activity at pH 7.0 in a sodium phosphate buffer; BglG167b is stable from pH 6.0 to 9.0; at pH 5.0 to 10.0, the enzyme stability decreased swiftly, while at pH 5.0 and 10.0 the enzyme activity decreased to 1.2% and 40.0% (Figure 2B). The enzyme is probably mesophilic and stable at a neutral pH range. These optimal conditions are consistent with the soil environment from which *Microbacterium* sp. Gsoil 167 was isolated. In addition, the near-neutral optimal pH and mild optimal temperature of BglG167b are similar to those of other ginsenoside-hydrolyzing GH3 from bacteria [22,23,27,29,30]. Although the optimum temperature of BglG167b for *p*NPGlc is 45 °C, the ginsenoside-conversion reaction occurred at 37 °C for the extension of stable transformation activity.

The effects of metal ions, EDTA, DTT, and SDS on BglG167b activity were also studied (Table S1). BglG167b activity was affected by 10 mM of β-mercaptoethanol, which is a well-known thiol group inhibitor. This suggested that sulfhydryl groups may be involved in the catalytic center of the enzyme. The enzyme was not affected by Na^+^, K^+^, Mn^2+^, and Mg^2+^. The chelating agent EDTA inhibits BglG167b activity, which indicated that divalent cations may be required for enzymatic activity. The enzyme activity appeared to be strongly inhibited in the presence of Ca^2+^, SDS, Zn^2+^, Co^2+^, and Cu^2+^. However, no dramatic positive effect on the activity of the BglG167b was found for the tested ions.

The substrate specificity of BglG167b was tested using 2.0 mM of *p*-nitrophenyl (*p*NP) and *o*-nitrophenyl (*o*NP)-glycosides with α and β configurations. The results showed that BglG167b was active against glucose moiety of *p*NP-β-d-glucopyranoside and *o*NP-β-d-glucopyranoside. It showed maximum activity towards *p*NP-β-d-glucopyranoside, 8.1% relative activity towards *p*NP-α-d-glucopyranoside and 22.9% relative activity towards *o*NP-β-d-glucopyranoside (Table S2).

### 2.5. Transformation Characteristics of BglG167b

**Figure 3:**
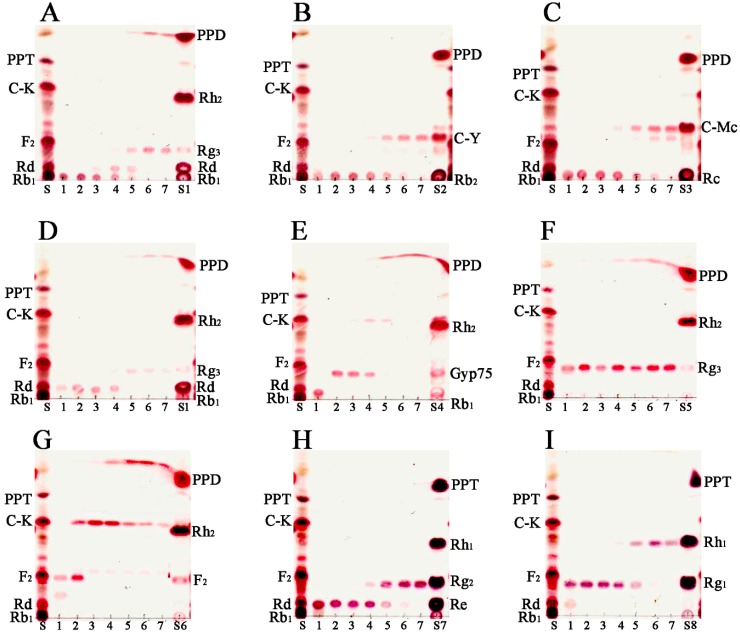
TLC analyses of time-course of ginsenoside bioconversion by BglG167b at an enzyme concentration of 1.0 mg/mL. (**A**) the transformation of Rb_1_; (**B**) the transformation of Rb_2_; (**C**) the transformation of Rc; (**D**) the transformation of Rd; (**E**) the transformation of gypenoside XVII; (**F**) the transformation of Rg_3_(*S*); (**G**) the transformation of F_2_; (**H**) the transformation of Re; and (**I**) the transformation of ginsenoside Rg_1_. Developing solvent: CHCl_3_–CH_3_OH–H_2_O (70:30:10, *v*/*v*, lower phase). Lane S, standards (Rb_1_, Rd, F_2_, C-K, PPT, PPD); Lane S1, standards (Rb_1_, Rd, Rg_3_(*S*), Rh_2_(*S*), PPD); Lane S2, standards (Rb_2_, C-Y, PPD); Lane S3, standards (Rc, C-Mc, PPD); Lane S4, standards (Rb_1_, GypXVII, GypLXXV, Rh_2_(*S*), PPD); Lane S5, standards (Rg_3_(*S*), Rh_2_(*S*), PPD); Lane S6, standards (F_2_, Rh_2_(*S*), PPD); Lane S7, ginsenoside standards (Re, Rg_2_(*S*), Rh_1_(*S*), PPT); Lane S8, ginsenoside standards (Rg_1_, Rh_1_(*S*), PPT); Lane 1, control; Lane 2, 5 min; Lane 3, 30 min; Lane 4, 3 h; Lane 5, 10 h; Lane 6, 24 h; Lane 7, 48 h.

For the verification of the bioconversion pathways of the nine ginsenosides (Rb_1_, Rb_2_, Rc, Rd, GypXVII, Rg_3_(*S*), F_2_, Re and Rg_1_) by BglG167b, the TLC analyses were performed at regular intervals. GST-BglG167b could clearly transform nine ginsenosides, as shown by the *R*_f_ values of the TLC analysis (Figure 3).

**Figure 4:**
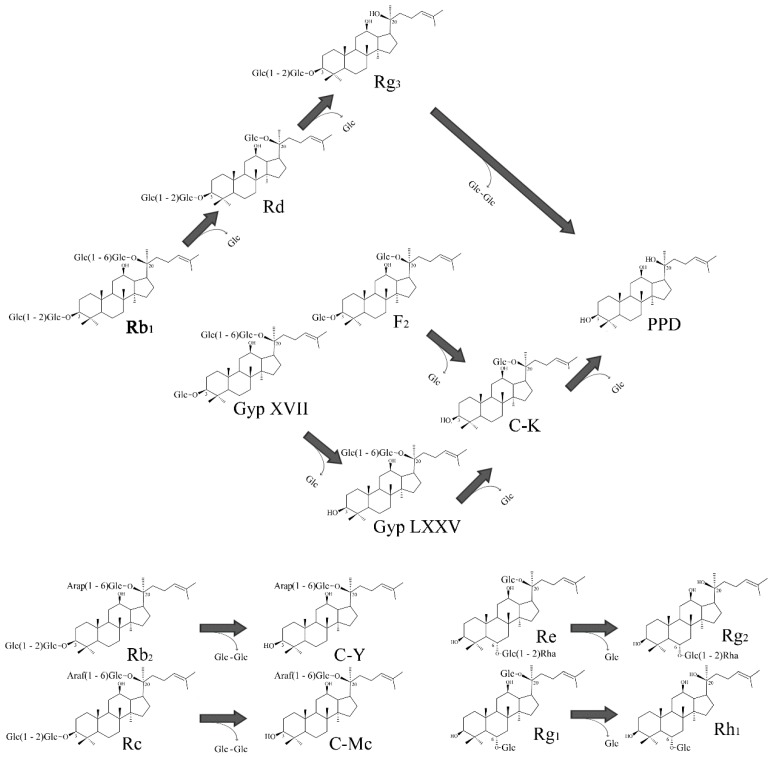
Transformation pathways of ginsenosides Rb_1_, Rb_2_, Rc, Rd, GypXVII, Rg_3_(*S*), F_2_, GypLXXV, Re, and Rg_1_ by recombinant BglG167b, respectively.

In PPD-type ginsenosides, Rb_1_, Rd, GypXVII, Rb_2_, and Rc have been converted by BglG167b. For Rb_1_ biotransformation, three types of metabolites (Rd, Rg_3_(*S*), PPD) were detected as time passed (Figure 4A). BglG167b successively hydrolyzed the outer and inner glucose moieties at position C20, and the inner glucose at position C3. BglG167b transformed all GypXVII into GypLXXV in 5 min, and continuously into C-K and PPD. The conversion speed of GypXVII was highest in all ginsenoside transformations of Bgl167b and it is the first enzyme in subfamily 5 which can hydrolyze GypXVII into GypLXXV. For F_2_ transformation, BglG167b produced C-K from F_2_, which means BglG167b prefers to hydrolyze glucose moiety at the C3 position than C20 when each position has one glucose moiety. According to the results of *p*NP and *o*NP substrates reactions, BglG167b could not hydrolyze arabinopyranoside and arabinofuranoside attached at the outer position of C20, but could hydrolyze two glucose moieties at C20. In PPT-type ginsenosides hydrolyzation, Re and Rg_1_ were transformed into Rg_2_(*S*) and Rh_1_(*S*), respectively, which means BglG167b cannot hydrolyze the attached sugars at C6, but glucose moieties at C3.

Summarizing the transformation pathways, the proposed biotransformation pathways by BglG167b for the ginsenosides are as follows: Rb_1_ → Rd → Rg_3_(*S*) → PPD; Rd → Rg_3_(*S*) → PPD; GypXVII → GypLXXV → C-K → PPD; F_2_ → C-K → PPD; Rb_2_ → C-Y; Rc → C-Mc; Re → Rg_2_(*S*); Rg_1_ → Rh_1_(*S*) via the stepwise hydrolysis of glucose moieties at the C20, and C3 positions of aglycon (Figure 4).

The metabolic pathways of ginsenoside Rb_1_, Rb_2_, and Rc hydrolysis by BglG167b are similar to β-glucosidases from *M. esteraromaticum* and *Flavobacterium johnsoniae*, which is attributed to their high amino acid sequence similarity in subfamily 5, and they can provide Rb_1_ → Rg_3_(*S*) conversions via hydrolysis of the outer and inner glucose at the C20 position of Rb_1_ (Figure S1) [26,27,31]. The transformation of GypXVII into GypLXXV has been found by β-glucosidases from *T. ginsenosidimutans*, *A. mirum*, and *S. keddieii* [22,23,25]. However, they simultaneously transform GypLXXV into C-K or PPD. On the other hand, the higher conversion ability of BglG167b makes it more efficient for the production of GypLXXV.

Previously, GypLXXV has been produced using microbial and enzymatic transformation, but the low concentration (less than 2.0 mg/mL), long transformation time (60 h), and further deglycosylation limited its application to the production of GypLXXV [22,32]. GypXVII has been produced at a gram-scale from major ginsenoside Rb_1_ using BgpA [20], and used for the substrate for the production of GypLXXV. BglG167b could efficiently transform GypXVII into GypLXXV at a higher concentration (5.0 mg/mL) and in a shorter period (9 h).

### 2.6. Scale-Up Production of GypLXXV and Purification

The bacterial cells that harbor pGEX-BglG167b were incubated further for 18 h at 22 °C after induction and were harvested via centrifugation when the culture reached an OD of 40 at 600 nm. One-hundred twenty-five grams (125 g) of wet cells were harvested and the pellets were resuspended in 10 volumes (*w*/*v*) of 100 mM sodium phosphate buffer (pH 7.0). The cells were disrupted via ultrasonication and the supernatant was used as crude enzymes for the biotransformation of the ginsenosides. 

**Figure 5:**
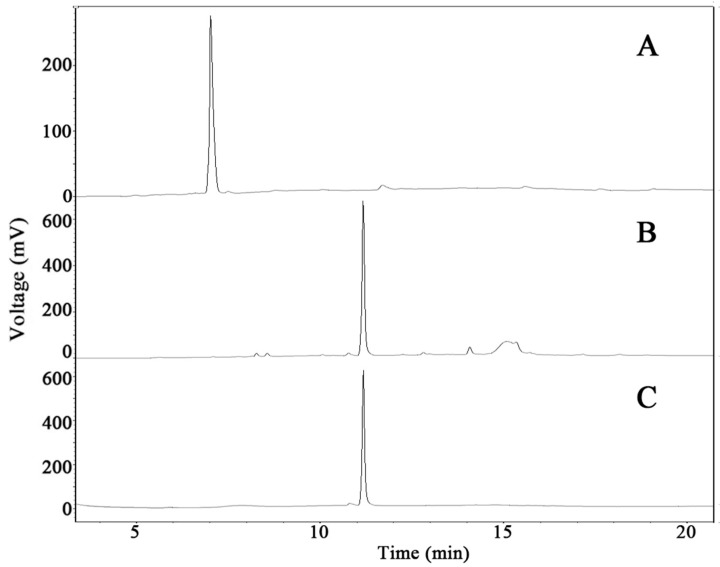
HPLC results of the production of GypLXXV from GypXVII by BglG167b; (A) substrate GypXVII; (B) the reaction mixture after nine-hour treatment with BglG167b; and (C) purified GypLXXV using Prep-HPLC.

Gram-scale GypXVII has been produced using recombinant BgpA from ginsenoside Rb_1_ (data not shown) [22]. The produced GypXVII (80.1% chromatography purity) was used as the substrate for the gram-scale production of GypLXXV. The enzyme reaction occurred using the crude recombinant BglG167b with GypXVII as the substrate with a concentration of 5.0 mg/mL. As shown in Figure 5, all GypXVII was converted to GypLXXV within 9 h (Figure 5B). Most of the produced GypLXXV was precipitated to form a solid, with a small quantity remaining dissolved in the supernatant.

In order to remove the free sugar, enzyme, and salt, the mixture was centrifuged at 5000 rpm for 15 min. After a purification step using column chromatography packed with HP20 resin, approximately 3 BVs of the 95% ethanol eluent was evaporated in vacuo in order to create 5.7 g of GypLXXV. Its chromatographic purity was 83.1% as determined via high-performance liquid chromatography (HPLC) (Figure 5). For the anti-cancer activity study, further purification was performed using Prep-HPLC. An ODS column is used as the stationary phase and 75% acetonitrile was used as the mobile phase. GypLXXV was eluted from 150 mL to 280 mL, and after recycling two times to remove impurity peaks, the compound with 97.8% purity was harvested. The recovery ratio through the biotransformation process using GypXVII to GypLXXV reached 69.6% during the entire bioprocess engineering.

Relatively abundant major ginsenosides were normally used as substrates for the production of minor ginsenosides, and we have demonstrated several enhanced productions of rare minor ginsenoside Rg_3_(*S*), F_2_, and Rg_2_(*S*) using various ginsenoside-transforming recombinant enzymes [31,33,34] from major ginsenosides. These various enzymatic methods were reported thus far for the preparation of minor ginsenosides as a result of their higher conversion efficiency, fewer by-products, superior environmental protection, and better stereo-specificity than physiochemical methods and microbiological methods [35,36,37]. Efforts have also been made to produce GypLXXV by deglycosylation of relatively abundant ginsenoside Rb_1_ [22,32]. Due to its rarity, GypXVII has not been used as a substrate for the preparation of GypLXXV. In this study, recombinant BgpA which has been cloned from *Terrabacter ginsenosidimutans* Gsoil 3082^T^ with high substrate tolerance (Rb_1_, 50 mg/mL) and efficiency (5 h), was used for the gram-scale GypXVII preparation [22]. Previously, GypXVII has been used for the production of F_2_ and CK [38,39]. However, no gram-scale production of GypLXXV, which is important for industrial application, has been achieved before, and the gram-scale production of GypLXXV showed a strong possibility for more large scale applications of BglG167b.

### 2.7. Cytotoxic Effect of GypLXXV on Cancer Cells

**Figure 6:**
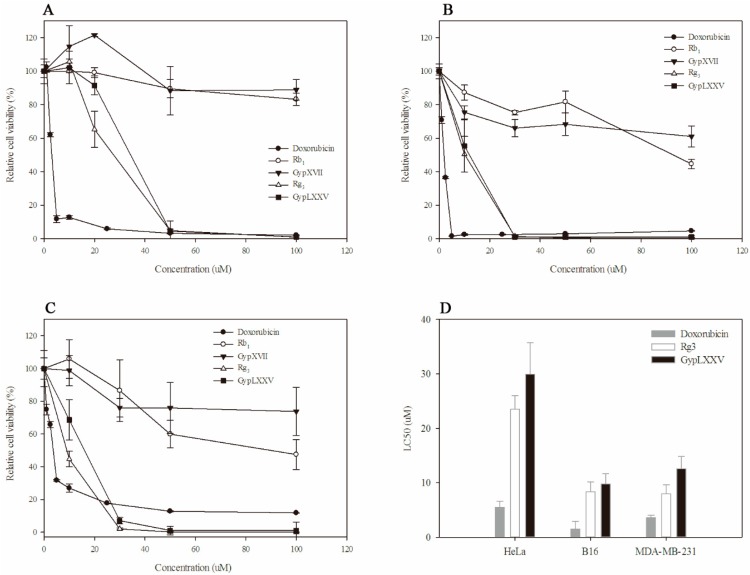
The anti-cancer effects of GypLXXV, Rg_3_(*S*), GypXVII and Rb_1_ on cell viability on (**A**) HeLa cells; (**B**) B16 cells; (**C**) MDA-MB231; and (**D**) LC50 comparison of doxorubicin, Rg_3_(*S*), and GypLXXV. Cancer cells were incubated with various concentrations of GypLXXV for 48 h.

In order to prove cytotoxic effects of GypLXXV on tumor cells, we compared the effect of Rb_1_, GypXVII, Rg_3_(*S*) and GypLXXV on cell viability using three cancer cell lines (HeLa (cervical cancer cell line), B16 (melanoma cell line), and MDA-MB231 (human breast cancer cell line)). Doxorubicin, a well-known chemotherapy medication used to treat cancer, was used as a positive control. The cancer cells were treated with 1.0–100 μM of pure doxorubicin, Rb_1_, GypXVII, Rg_3_(*S*), and GypLXXV for 48 h (Figure 6A–C). According to MTT assay results, treatment with Rg_3_(*S*) and GypLXXV reduced proliferation in a dose-dependent manner (Figure 6A–C). After 48 h of treatment, almost all cancer cells were inhibited by GypLXXV and Rg_3_(*S*) at 50 μM. The LC_50_ of doxorubicin was lower than Rg_3_(*S*) and GypLXXV on three cancer cells (Figure 6D). Though, as compared with LC50 doxorubicin and Rg_3_(*S*) (Figure 6D), GypLXXV showed considerable anti-proliferate activities higher than Rb_1_ and GypXVII, and 63–78% of to ginsenoside Rg_3_(*S*), which has shown strong anti-cancer effects in vitro and in vivo in accordance with many studies [40].

Anticancer effects of minor ginsenosides (F_2_, Rg_3_(*S*), Rh_2_(*S*), CK, and PPD) which were the deglycosylated form of ginsenoside Rb_1_, have been studied, and these minor ginsenosides showed strong activity against various cancer cell lines [41,42]. Among them, Rg_3_(*S*) appeared on the market and has been applied to clinical therapy. GypLXXV has two glucose moieties attached at the C20 position of aglycon, whereas Rg_3_(*S*) has two glucose moieties at C3 (Figure 4). The LC_50_ of GypLXXV showed similar anti-cancer effects with Rg_3_(*S*) (Figure 6D), and higher than Rb_1_ and GypXVII, which have more attached glucose moieties (Figure 6A–C). It is the first report of the anti-cancer activity of GypLXXV, and the patent of the anti-cancer activity of GypLXXV against HeLa cells has been registered by this team [43]. These results demonstrate that deglycosylation contributes to improved anticancer activity, and the mechanism of increased anticancer effects requires further structure-related approaches.

## 3. Materials and Methods

### 3.1. Materials

Ginsenoside standards that are over 98% purity, such as Rb_1_, Rb_2_, Rc, Rd, Rg_3_(*S*), Rh_2_(*S*), F_2_, compound K (C-K), protopanaxadiol (PPD), Rg_1_, Re, Rg_2_(*S*), Rh_1_(*S*), and protopanaxatriol (PPT), were purchased from (Zelang Medical Technology Co., Ltd., Nanjing, China). HPLC grade acetonitrile was obtained from Merck (Darmstadt, Germany). GypXVII, GypLXXV, and ginsenosides compound O (C-O), compound Y (C-Y), compound Mc_1_ (C-Mc_1_), and compound Mc (C-Mc), were prepared as described by Cui et al. [23]. 5-bromo-4-chloro-3-indolyl β-d-glucopyranoside (X-Glc) was purchased from Wako Co. Ltd. (Tokyo, Japan). The other chemicals used were a minimum of analytical reagent grade. *Microbacterium* sp. Gsoil 167, was isolated from a soil sample of a ginseng field, Pocheon Province, South Korea, and cultivated on R2A agar (Becton, Dickinson and Company, Franklin Lakes, NJ, USA) under aerobic conditions at 30 °C and used for the gene cloning experiment. *Escherichia coli* BL21 (DE3), and pGEX 4T-1 plasmid (GE Healthcare, Chicago, IL, USA) were used as host and expression vector sources, respectively. The *E. coli* cells harboring the BglG167b-containing recombinant plasmid for protein expression was cultivated in a Luria-Bertani (LB) medium supplemented with ampicillin (100 mg/L).

### 3.2. Fosmid Library Construction and Fosmid Sequencing

A CopyControl^™^ Fosmid Production kit (Epicentre Technologies, Madison, WI, USA) was used to isolate the ginsenoside-hydrolyzing glycosidase genes from *Microbacterium* sp. Gsoil 167. A fosmid library was constructed following the manufacturer’s protocol. Infected *E. coli* was transferred onto X-Glc (5-bromo-4-chloro-3-indolyl β-d-glucopyranoside) LB plates supplemented with 12.5 μg/mL chloramphenicol and then incubated at 37 °C for 16 h. The blue color colonies were selected as putative ginsenoside-hydrolyzing candidates. After confirmation of the ginsenoside-hydrolyzing activity by a TLC assay, one clone was selected for fosmid sequencing. Fosmid DNA was purified and sequenced by Macrogen Co. Ltd. (Seoul, Korea). The final sequences assembly procedure was conducted by the SeqMan program in the DNASTAR package (DNASTAR, Madison, WI, USA), which yielded a 9.4-kb contig.

### 3.3. Phylogenetic Analysis of BglG167b

A homology search was performed with BLAST program provided by NCBI database (blast.ncbi.nlm.nih.gov). Sequences of the characterized glycosyl hydrolases were obtained from the CAZY database [44], and multiple alignments were performed using the CLUSTAL_X program [45]. Gaps were modified in the BioEdit program [46], and evolutionary distances were calculated according to the Kimura two-parameter model [47]. A phylogenetic tree was constructed using the neighbor-joining method in the MEGA5 program, with bootstrap values based on 1000 replicates [48].

### 3.4. Molecular Cloning, Expression, and Purification of Recombinant BglG167b

The fosmid DNA sequence was analyzed using the ORF Finder program on the NCBI website (www.ncbi.nlm.nih.gov/gorf). Predicted ORFs were subjected to similarity searches using the BLAST program, which identified two putative open reading frames (ORFs) for β-glucosidases. One of them, BglG167b, which belonged to GH3 and consisted of 803 predicted amino acids, showed ginsenoside-transforming activities. To clone the gene encoding BglG167b, PCR amplification was conducted using primers containing BamHI and XhoI (BglG167b) restriction sites, respectively (bolded). The primer sequences were as follows: bglC167bF 5′-GGT TCC GCG T**GG ATC C**AA CGC CCG CCC GTC CGA TTC CAC C-3′ and bglC167bR 5′-GAT GCG GCC G**CT CGA G**TC ATG CCT GCT CCC AGT CCA CCG A-3′. The amplified DNA fragment obtained from the PCR was purified and inserted into the pGEX 4T-1 using an EzCloning Kit (Enzynomics Co. Ltd., Daejeon, Korea). The recombinant pGEX-BglG167b was transformed into *E. coli* BL21 (DE3). The recombinant plasmid harboring *E. coli* BL21 (DE3) was grown in an LB-ampicillin medium at 37 °C until the culture reached an OD_600_ of 0.6, and then the recombinant protein expression was induced through 0.1 mM isopropyl-β-d-thiogalactopyranoside (IPTG). The bacterial cells were incubated for a further 18 h at 18 °C and harvested via centrifugation at 13,000 rpm for 15 min at 4 °C. The cells were washed twice with a solution consisting of 50 mM sodium phosphate, 5 mM EDTA, and 1% Triton X-100 (pH 7.0); then, they were resuspended in 50 mM sodium phosphate (pH 7.0). The cells were lysed via ultrasonication (Vibra-cell, Sonics and Materials Inc., Newtown, CA, USA). The insoluble debris was removed via centrifugation at 13,000 rpm for 15 min at 4 °C in order to obtain the crude cell extract. The GST-tagged BglG167b was purified using the GST bind agarose resin according to the manufacturer’s protocol (Elpisbiotech Co. Ltd., Daejeon, Korea). The purity of the protein was assessed using 10% SDS-PAGE and an EZ-Gel staining solution (Daeillab Co. Ltd., Seoul, Korea).

### 3.5. Effect of pH, Temperature, Metal Ions, and Chemical Reagents on Enzyme Activity

The specific activity of purified BglG167b was determined using *p*NP-β-d-glucopyranoside (*p*NPGlc) as a substrate in pH 7.0, 50 mM sodium phosphate buffer at 37 °C. Reactions were performed for 10 min by the addition of Na_2_CO_3_ (final concentration, 500 mM), and the release of *p*-nitrophenol was measured directly using a microplate reader at 405 nm (Bio-Rad Model 680; Bio-Rad., Hercules, CA, USA). One unit of activity was defined as the amount of protein required to generate 1 μmol of *p*-nitrophenol per min. Protein concentrations were determined using the BCA protein assay protocol (Pierce, Rockford., IL, USA). All assays were performed in triplicate.

The effect of pH on enzymatic activity was determined as described [22]. The pH stability of recombinant BglG167b was determined by quantifying enzymatic activity after incubation in each buffer for 24 h at 4 °C. The effect of temperature was tested by incubating the enzyme at various temperatures ranging from 4 °C to 65 °C at the optimum pH for 10 min containing 2.0 mM *p*NPGlc. The thermostability of the enzyme was determined by incubating the enzyme in 50 mM sodium phosphate buffer (pH 7.0) for 2 h at different temperatures. After chilling the sample on ice for 10 min, activity was determined using *p*NPGlc.

The effects of metals and other chemicals on BglG167b activity were also determined. BglG167b activity was tested in the presence of 1 or 10 mM (final concentration) of CaCl_2_, CoCl_2_, CuCl_2_, KCl, MgCl_2_, NaCl, ZnCl_2_, dithiothreitol (DTT), EDTA, or SDS for 30 min at 25 °C. The remaining activity was determined and expressed as a percentage of the activity obtained in the absence of the compound.

Substrate preference was determined using 2.0 mM chromogenic *o*-nitrophenyl (*o*NP) and *p*-nitrophenyl (*p*NP) as surrogate substrates (all from Sigma) at 37 °C for 10 min, with one unit being defined as the release of 1 μmol *p*-nitrophenol or *o*-nitrophenol per min.

### 3.6. Biotransformation Activity of Ginsenosides Using BglG167b

The initial biotransformation experiments using the major ginsenosides Rb_1_ and Re as substrates revealed that the GST-fused enzyme does not affect the activities of BglG167b. Therefore, the fusion protein (GST-BglG167b) was used to determine the specificity and selectivity of the enzymes for the hydrolysis of the glucose moieties attached at the C3 and C20 positions in the seven PPD ginsenosides and two PPT ginsenosides. The purified enzyme at a concentration of 1.0 mg/mL in 50 mM of sodium phosphate buffer (pH 7.0) were reacted with an equal volume of Rb_1_, Rb_2_, Rc, Rd, GypXVII, Rg_3_(*S*), F_2_, Re, and Rg_1_ solution at a concentration of 2 mg/mL in 50 mM of sodium phosphate buffer (pH 7.0) at 37 °C. The samples were taken at regular intervals and analyzed via TLC after pretreatment (see analytic methods). For TLC analysis, an equal volume of water-saturated *n*-butanol was added to stop the reaction, and the reactant present in the *n*-butanol fraction was determined.

### 3.7. Gram-Scale GypLXXV Production Using Recombinant BglG167b

#### Preparation of Recombinant BglG167b Using High Cell Density Culture

For the production of the recombinant BglG167b, the LB medium supplemented with ampicillin (100 mg/mL) was used to cultivate the *E. coli* BL21 (DE3) harboring *pGEX-*BglG167b in a 10 L tank reactor (Hanil science Co., Seoul, Korea) with a 5 L working volume at a 500 rpm stirring speed. The pH value of the medium was adjusted to 7.0 by the addition of (29%, *v*/*v*) of NH_4_OH and/or (37%, *v*/*v*) HCl. The culture was incubated at 37 °C until the culture reached an OD600 of 3.0.

Cells harboring pGST-BglG167b were induced at 18 °C for 18 h and were then harvested via centrifugation at 5000 rpm for 20 min at 4 °C. Cell pellets were resuspended in 10 volumes (*w*/*v*) of 50 mM sodium phosphate buffer (pH 7.0) and disrupted by sonication, and the supernatant was used as the crude enzyme for ginsenoside biotransformation.

GypXVII had been produced from Rb_1_ using recombinant BgpA (17). The scaled-up biotransformation of GypXVII into GypLXXV was performed in a 5.0 L glass bottle (2.0 L working volume) under optimal conditions (shaking 200 rpm for 8 h at pH 7.0 and 37 °C). The reaction consisted of crude recombinant 1.0 L BglG167b, 10 g of GypXVII in 1.0 L of 50 mM sodium phosphate buffer. Samples were collected at regular intervals and HPLC was used to monitor the biotransformation of GypXVII to GypLXXV, respectively.

### 3.8. Purification of GypLXXV

Following the 2.0 L reaction of GypXVII with BglG167b, the mixture was cooled at 4 °C and centrifuged at 5000 rpm for 20 min. The supernatants and precipitates were processed separately in order to purify the samples. The precipitate was also dissolved in 5.0 L of 70% ethanol solution twice and filtered using a filter paper (Advantec., Tokyo, Japan). The ethanol extracts were combined with the supernatant and adjusted to be a 45% ethanol solution. The column chromatography (3170 (L) × 128 (D) mm; Doointech, Korea) packed with HP20 resin (Mitsubishi Chemical Corp., Tokyo, Japan) was adopted in order to remove the impurities, except the ginsenosides. The free sugar molecules and unwanted hydrophilic compounds from the HP-20 that were adsorbed in beads were washed with three bed volumes (BV) of water, and the adsorbed ginsenosides were finally eluted using 3 BVs of 95% ethanol. The ethanol eluent was evaporated in vacuo. The resulting powder was dissolved in 100% methanol and analyzed via HPLC.

GypLXXV was further purified using preparative HPLC (JAL Recycling Preparative HPLC LC-9210II NEXT, Japan Analytical Industry Co., Tokyo, Japan). The preparative HPLC run was performed using a pre-packed column (JAIGEL-ODS-AP-L, 20 mm i.d. × 500 mm) purchased from Japan Analytical Industry Co. (Tokyo, Japan). The mobile phase was 85% acetonitrile, and the flow rate was 7.0 mL/min. The sample solution was made by dissolving a sufficient quantity of crude GypLXXV in 85% acetonitrile to give a final concentration of 30 mg/mL and 10 mL was loaded for the purification. GypLXXV was returned to the column after passing through the detector two times to achieve a higher purification. The elutionfrom 70 min to 77 min was collected, evaporated and analyzed by HPLC.

### 3.9. Cell Culture

Cancer cells were maintained in Dulbecco’s Modified Eagle’s Medium (DMEM) with 10% FBS, 2 mmol/L glutamine, 100 U/mL penicillin, and 100 mg/mL streptomycin at 37 °C in a humidified 5% CO_2_ atmosphere. Appropriate amounts of the resulting GypLXXV in DMSO were added to the culture medium at the indicated final concentrations.

### 3.10. MTT Assay

The in vitro chemosensitivity was measured by dimethyl thiazolyl-2,5-diphenyltetrazolium bromide (MTT) assay (CellTiter 96^®^ Non-Radioactive Cell Proliferation Assay kit, Promega, Madison, WI, USA). Briefly, Cancer cells were inoculated into 96-well plates in 100 μL medium (10^4^ cells) per well and allowed to attach and grow for 24 h. To determine the anti-proliferative effect of GypLXXV, Rg_3_(*S*), Rb_1_ and GypXVII, various concentrations of ginsenosides diluted with the FBS-free medium were added into the wells. Then the cells were exposed to drugs for 48 h at 37 °C, after which, MTT was added to each well and cultured for an additional 4 h. The formed formazan was dissolved in 100 μL of solubilization/stop solution after aspirating the culture medium. The plates were shaken mechanically for 5 min and incubated for 1 h. After shearing for each well the optical density was immediately read on a microplate reader at a wavelength of 595 nm. Results are expressed as LC_50_ which was analyzed by GraphPad Prism 5 program.

### 3.11. Analytic Methods

#### 3.11.1. Thin Layer Chromatography (TLC) Analysis

The thin layer chromatography (TLC) was performed using 60F_254_ silica gel plates (Merck & Co., Munchen, Germany) with CHCl_3_–CH_3_OH–H_2_O (70:30:10, lower phase) as the solvent. The spots on the TLC plates were identified through comparisons with standard ginsenoside after visualization was made by spraying 10% (*v*/*v*) H_2_SO_4_, followed by heating at 110 °C for 5 min.

#### 3.11.2. High-Performance Liquid Chromatography (HPLC) Analysis

The HPLC analysis of the ginsenosides was performed using an HPLC system (Younglin Co. Ltd., Anyang-si, Korea). The separation was performed on a Prodigy ODS(2) C_18_ column (5 μm, 150 × 4.6 mm i.d.; Phenomenex Inc., Torrance, CA, USA) with a guard column (Eclipse XDB C_18_, 5 μm, 12.5 × 4.6 mm i.d.). The mobile phases were A (acetonitrile) and B (water). The gradient elution started with 32% solvent A, and was changed to the following: from 0–8 min, A was increased from 32 to 65%; from 8–12 min, A was increased from 65 to 100%; from 12–15 min, A was constant at 100%; from 15–15.1 min, A was decreased from 100 to 32%; from 15.1–25 min, A was constant at 32% The flow rate was 1.0 mL/min, and the detection was measured by monitoring the absorbance at 203 nm and with an injected volume of 25 μL.

### 3.12. Nucleotide Sequence Accession Numbers

The sequence for the BglG167b gene from *Microbacterium* sp. Gsoil 167 was deposited into GenBank/EMBL/DDBJ under accession numbers JX960414.

## 4. Conclusions

In summary, we have cloned and characterized ginsenoside-transforming β-glucosidase belonging to the glycoside hydrolases superfamily 3 from *Microbacterium* sp. Gsoil 167 from the ginseng-cultivating soil. This had optimum reaction conditions at 37 °C and pH 7.0. BglG167b could convert various ginsenosides (Rb_1_, Rb_2_, Rc, Rd, GypXVII, Rg_3_(*S*), F_2_, Re, and Rg_1_) through the selective hydrolysis of only the glucose moieties. Among them, this enzyme efficiently hydrolyzes one inner glucose moiety at the C3 position of PPD type ginsenosides (F_2_ and GypXVII). The recombinant BglG167b was applied to produce GypLXXV on the gram-scale and scale-up of production using 10 g of the GypXVII resulting in 5.7 g of GypLXXV with 97.8% chromatography purity and 69.6% recovery. The anti-cancer activity of this compound was primarily determined in vitro. GypLXXV showed strong cancer cell growth inhibition of cancer cells, and the activity is similar with the well-known anti-cancer ginsenoside-Rg_3_(*S*) which showed its possibility for the application. Therefore, the properties of BglG167b could be a useful tool in the research and production of GypLXXV in the cosmetics, functional food, and pharmaceutical industries.

## Supplementary Materials

Supplementary materials are available online.
