# Supplementary material for: Enhanced Production of Gypenoside LXXV Using a Novel Ginsenoside-Transforming β-Glucosidase from Ginseng-Cultivating Soil Bacteria and Its Anti-Cancer Property
Source: Molecules. 2017 May 19;22(5):844. doi: 10.3390/molecules22050844 (PMC6153937; doi:10.3390/molecules22050844)
Supplement: Supplementary file 1 [file molecules-22-00844-s001.pdf]

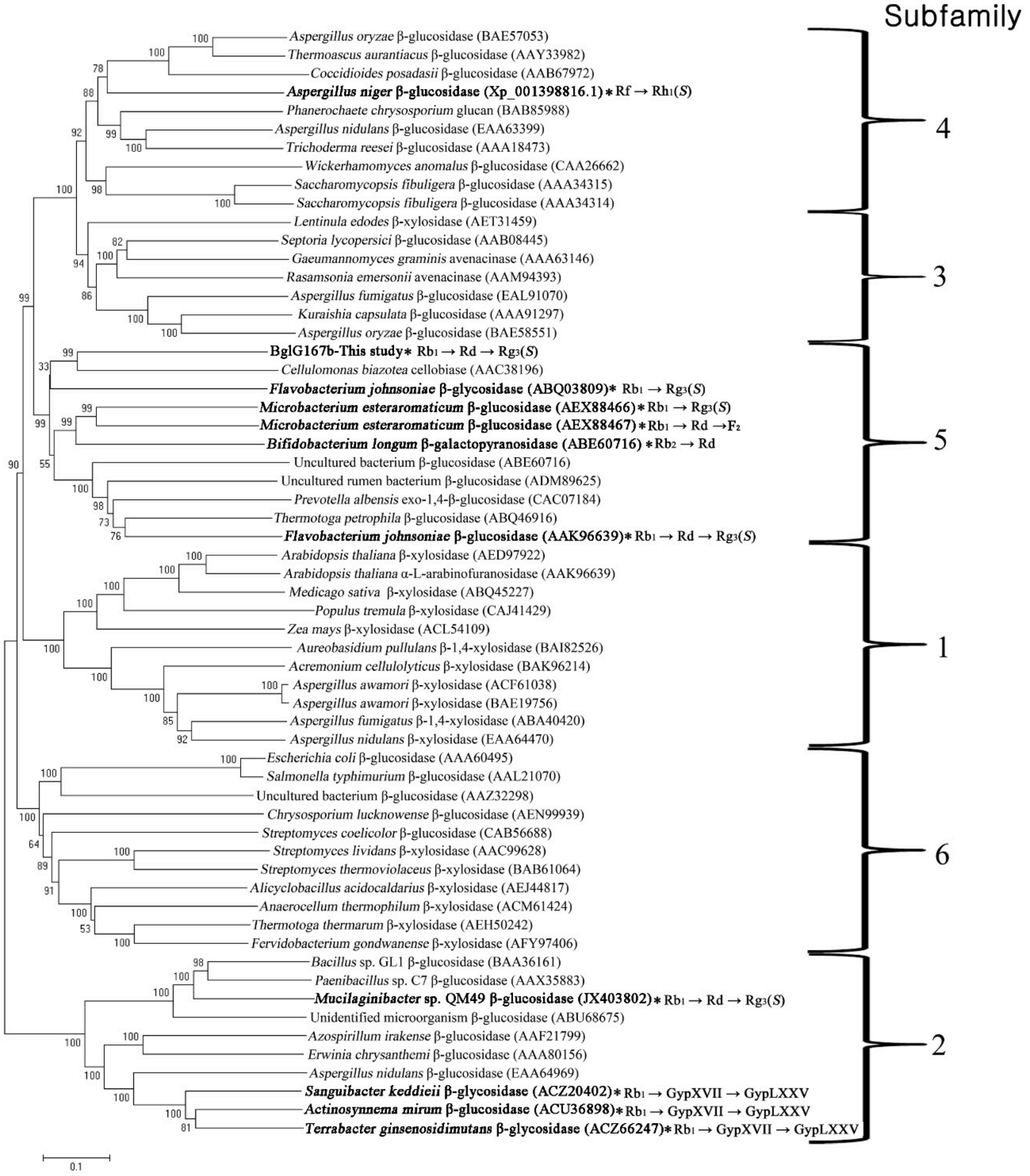

Fig. S1. Phylogenetic analysis of BglG167b in characterized glycoside hydrolases in family 3. Amino acid sequences were obtained from the NCBI database and CAZy database (accession numbers are indicated on the tree). This tree was made using the neighbor-joining method with a Kimura two-parameter distance matrix and pairwise deletion.

Table S1. Effects of metal ions and chemical agents on the activity of purified recombinant BglG167b.

Metal ion and reagent	Relative activity ± SD (%)	
	1 mM	10 mM
NaCl	100.0±6.3	94.4±1.6
KCl	104.9±1.7	95.7±3.3
$MgCl_2$	90.9±2.8	93.9±1.4
$CaCl_2$	94.4±5.7	61.0±4.7
$ZnCl_2$	44.6±6.0	26.3±9.6
$CoCl_2$	93.1±5.4	38.2±4.5
$CuCl_2$	13.8±1.0	14.0±1.0
SDS	33.8±9.0	19.9±0.6
EDTA	81.0±3.7	28.9±2.8
DTT	85.9±1.1	46.9±5.9
Control	100.0	100.0

Table. S2. Relative activity of purified recombinant BglG167b towards various chromogenic substrates as measured by *o*NP or *p*NP release at 37°C. ND: not determined.

	Substrate[a]	Relative activity ± SD (%)[b]
1	*p*NP-*α*-D-glucopyranoside	8.1 ± 2.6
2	*p*NP-*α*-D-mannopyranoside	ND
3	*p*NP-*α*-D-xylopyranoside	ND
4	*p*NP-*α*-L-arabinofuranoside	ND
5	*p*NP-*α*-L-arabinopyranoside	ND
6	*p*NP-*α*-L-rhamnopyranoside	ND
7	*p*NP-*β*-D-fucopyranoside	ND
8	*p*NP-*β*-D-galactopyranoside	ND
9	*p*NP-*β*-D-glucopyranoside	100.0 ± 7.2
10	*p*NP-N-acetyl--*β*-D-glucosaminide	ND
11	*p*NP-*β*-D-mannopyranoside	ND
12	*p*NP-*β*-D-xylopyranoside	ND
13	*p*NP-*β*-L-arabinopyranoside	ND
14	*o*NP-*α*-D-galactopyranoside	ND
15	*o*NP-*β*-D-fucopyranoside	ND
16	*o*NP-*β*-D-galactopyranoside	ND
17	*o*NP-*β*-D-glucopyranoside	22.9 ± 0.9

[a]Final concentration, 2.0 mM.

[b]Activity toward *p*NP-*β*-D-glucopyranoside was set as 100%.

REFERENCES

Kimura, M. (1983). The neutral theory of molecular evolution. Cambridge University Press, Cambridge.
